# Boosting hyperalignment performance with age-specific templates

**DOI:** 10.7554/eLife.110566

**Published:** 2026-08-05

**Authors:** Yuqi Zhang, Maria Ida Gobbini, James V Haxby, Ma Feilong

**Affiliations:** 1 Dartmouth College Hanover United States; 2 University of Bologna Bologna Italy; 3 University of South Carolina Columbia United States; Washington University in St. Louis United States; Radboud University Nijmegen Netherlands

**Keywords:** fMRI, hyperalignment, brain aging, individual differences, functional connectivity, Human

## Abstract

Hyperalignment aligns individual brain activity and functional connectivity patterns to a common, high-dimensional model space, resolving idiosyncrasies in functional–anatomical correspondence and revealing shared information encoded in fine-grained spatial patterns. Given that the brain undergoes significant developmental and functional changes over the lifespan, certain features in brain functional organization may be more prominent in certain age groups than others. In this study, we examined whether age-specific functional templates, compared with a canonical template, could enhance alignment accuracy across diverse age groups. We used the Cambridge Centre for Ageing and Neuroscience (Cam-CAN) dataset (18–87 years old) to build age-specific templates and tested their performance in young and old brains in both the Cam-CAN dataset and the Dallas Lifespan Brain Study dataset (20–90 years old). We found the congruent age-specific template outperforms the incongruent template for various analyses, including inter-subject correlation of hyperaligned connectivity profiles and predictions of individualized connectomes and brain responses to the movie. The results are consistent across both datasets. This work enhances our understanding of age-related differences in brain function, highlights the benefits of age-specific templates to refine hyperalignment model performance, and may contribute to the development of age-sensitive diagnostic tools and interventions for neurological disorders.

## eLife digest

Brains can implement the same function in different ways. For example, across individuals, the same anatomical location may serve different roles, and the same functional region may vary in size, location, and shape. As a result, directly comparing anatomical locations across brains is like comparing apples to oranges.

To address this issue, researchers use a method called hyperalignment, which aligns brains functionally before analysis. This technique establishes correspondence across individuals by arranging their brains to a common template, allowing researchers to study both commonalities and individual differences in brain function.

However, certain brain features change over time and can be more prominent in some age groups. Zhang et al. sought to determine whether age-specific functional templates would be more accurate across age groups.

The researchers used preexisting fMRI data from people aged 18 to 87 to build age-specific templates and tested their performance on additional datasets. They found that hyperalignment templates derived from the same age group consistently outperformed those from other age groups by better capturing the functional brain organisation of individuals within the same age group and predicting brain responses. However, performance declined as the difference between a participant’s age and the template age increased.

In the long term, these findings may improve our ability to study the neural mechanisms of different populations, including individuals of different ages and those with neurological or psychiatric conditions. Population-specific templates could make brain mapping more sensitive, enabling better detection of group-specific functional features. While this study focuses on age differences, future work examining other populations will help establish the importance of building tailored templates for various conditions.

## Introduction

Information encoded in the cortex can be decoded from fine-grained patterns of cortical activity via multivariate pattern analysis of fMRI data (Haxby et al., 2001; Haxby et al., 2014). However, there is considerable variability across individual brains when encoding the same information (Cox and Savoy, 2003). Traditional approaches to brain alignment often fail to capture the fine-grained functional correspondence between brains due to topographic variability. Hyperalignment is a computational approach for modeling how the brain encodes shared information across individuals, despite individual variability in cortical topographies (Haxby et al., 2011; Haxby et al., 2020). Hyperalignment can be trained using either brain responses (Haxby et al., 2011; Guntupalli et al., 2016) or functional connectivity (Guntupalli et al., 2018), and it captures both shared coarse- and fine-scale information encoded in the brain. This approach also enhances the reliability of individual differences by affording analysis of differences in the fine-grained structure of the functional connectome (Feilong et al., 2018; Feilong et al., 2021).

Given that the brain undergoes substantial developmental, structural, and functional changes across the lifespan, brain functional organization may demonstrate age-specific features that are not prominent in other age groups. These age-related variations can impact the alignment of neural data across individuals. Consequently, the templates used in human connectomics may be significantly influenced by the age of the individuals used to build the templates. In this study, we investigate whether the incorporation of age-specific templates enhances the performance of hyperalignment models. Examining this aspect could not only improve hyperalignment accuracy across diverse age groups but also inform age-sensitive intervention strategies. Individual differences in the brain arise from both aging and neurological diseases and brain injuries. This knowledge also could open new pathways for creating more effective diagnostic tools for neurological disorders (Anderson et al., 2021; Anderson et al., 2024).

We investigated this problem by developing age-specific hyperalignment templates using the Cambridge Centre for Ageing and Neuroscience (Cam-CAN) dataset and evaluating their performance with three indices: (1) inter-subject correlation (ISC) of connectomes, (2) prediction accuracy of individualized connectomes, and (3) prediction accuracy of individualized brain responses to the movie. Across all three analyses, we found consistent advantages of congruent age-specific templates (i.e., those constructed using data from the same age group) over incongruent templates. Together, these results demonstrate the importance of accounting for age-specific features of brain functional organization when applying functional alignment methods.

## Results

We evaluated the performance of the templates using three metrics: (1) ISC of functional connectivity in the common template space, where higher ISCs indicate better alignment of connectivity in the template space; (2) similarity between model-predicted and measured connectomes in each participant’s native anatomical space; and (3) similarity between model-predicted and measured neural responses to the movie, where higher similarity suggests the template contains more relevant information and better predicts individualized connectomes and movie responses. Across all metrics, we found that congruent age-specific templates outperformed age-incongruent templates.

### Inter-subject correlation

**Figure 1.**
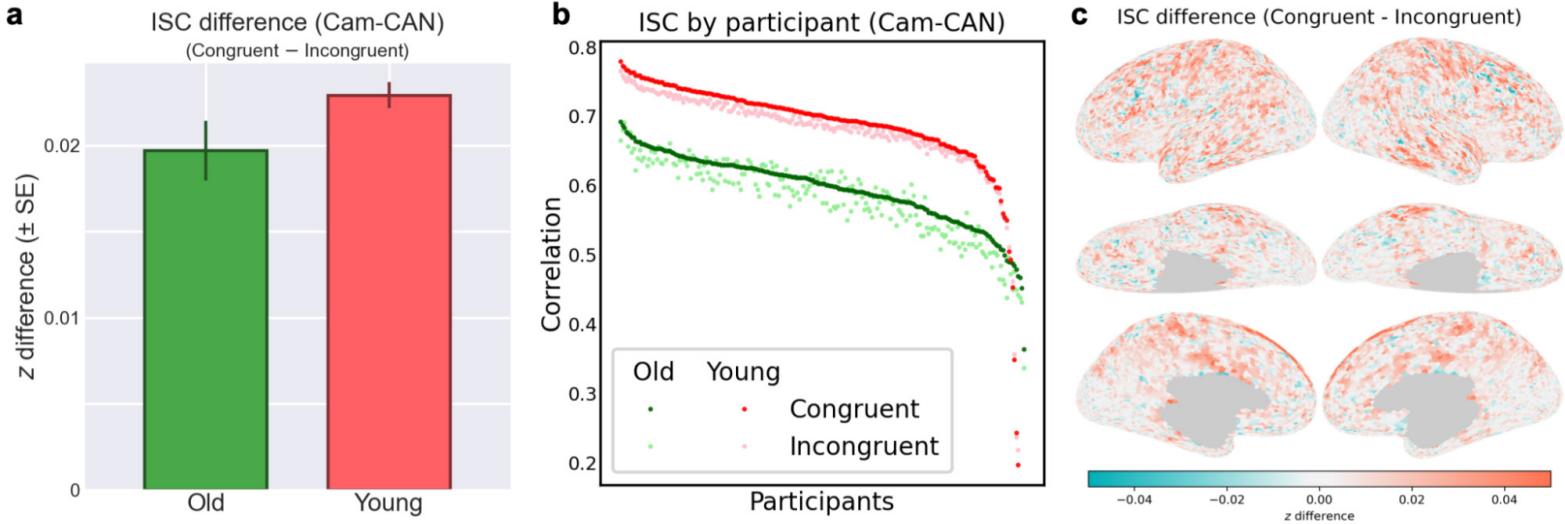
Inter-subject correlation (ISC) results (Cam-CAN). (**a**) The average *z* (± SD) for the old group was 0.6897 ± 0.0759 for congruent templates, and 0.6700 ± 0.0839 for incongruent templates. The mean ISC difference (± SE) was 0.0197 ± 0.0017, *t*(212) = 11.33, Cohen’s *d* = 0.7766, and p < 10^–22^. The average *z* (± SD) for the young group was 0.8566 ± 0.1151 for congruent templates, and 0.8337 ± 0.1069 for incongruent templates. The mean ISC difference (± SE) was 0.0229 ± 0.0008, *t*(209) = 30.09, Cohen’s *d* = 2.0765, and p < 10^–77^. (**b**) Scatter plot of each participant’s average ISC value derived from different congruent and incongruent templates. (**c**) Topographic distribution of ISC difference across different brain regions. Figure 1—source data 1.Source data for Inter-subject correlation (ISC) results (Cam-CAN).

We first evaluated the performance of templates constructed from both young and old age groups on ISCs of connectomes in the common model template space. We computed the *z*-difference (difference in Fisher *z* transformed correlations) between post-alignment ISC values derived from congruent and incongruent templates (Figure 1a). Hyperalignment based on congruent age-specific templates yields higher ISCs in both age groups, with a more pronounced effect in the young group. Breaking down the data by participant, we found that most participants (97.6% in the young group, 74.2% in the old group) exhibited higher ISCs when comparing the results for congruent and incongruent templates (Figure 1b). We also evaluated the ISC performance of an intermediate middle-aged cohort using a middle-aged template; these results are provided in Appendix 1—figure 4. The topography of mean ISC differences displayed in Figure 1c illustrates that congruent templates perform better in the frontal, temporal, and parietal lobes. The ISC for the young group was consistently higher than that for the old group for both congruent and incongruent templates. This reflects, at least in part, differences in data quality between the two age groups (Appendix 1—figure 2).

### Predicting connectomes

Higher ISCs can arise from both (1) better alignment across participants and (2) filtering out noise during hyperalignment transformations. Though it is unlikely that our ISC results were driven by reduced noise, to rule out the possibility, we assessed how well different templates predict each participant’s connectome.

For each individual, we calculated the correlation between the predicted connectome, generated using young and old training group hyperalignment templates calculated with rest and smt fMRI data, and the actual connectome, calculated using movie-watching fMRI data. A higher correlation indicates a more accurate prediction, which means better performance of the hyperalignment template.

**Figure 2.**
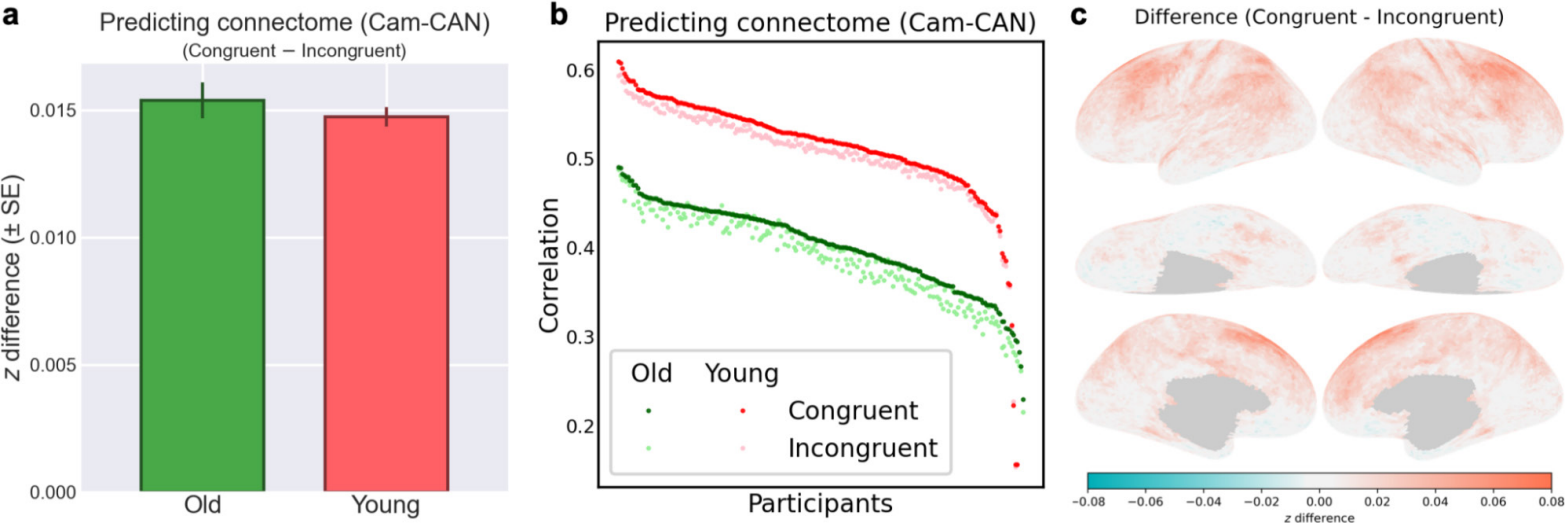
Prediction performance comparison between congruent and incongruent templates. (**a**) The average *z* (± SD) for the old group was 0.4279 ± 0.0558 for congruent templates, and 0.4126 ± 0.0597 for incongruent templates. The mean difference of predicted connectome (± SE) was 0.0154 ± 0.0007, *t*(212) = 21.91, Cohen’s *d* = 1.5014, and p < 10^–55^. The average *z* (± SD) for the young group was 0.5716 ± 0.0779 for congruent templates, and 0.5568 ± 0.0743 for incongruent templates. The mean difference of predicted connectome (± SE) was 0.0147±0.0004, *t*(209) = 39.07, Cohen’s *d* = 2.6963, and p < 10^–97^. (**b**) Scatter plot of each participant’s average correlation value derived from different congruent and incongruent templates. (**c**) Topographic correlation difference across different brain regions. Figure 2—source data 1.Source data for prediction performance comparison between congruent and incongruent templates (Cam-CAN).

We compared the performance of congruent templates (built from the same age group) and incongruent templates (built from a different age group). For each participant in the test sets of both age groups, we calculated the predicted connectome using 20 templates—10 from each age group. We then computed the correlation for each participant and averaged the individual results across templates in the same age group. Figure 2a shows the *z*-difference between correlations calculated using congruent and incongruent templates for each participant in both groups. The results clearly indicate that predictions using congruent templates are more accurate than those using incongruent templates. Examining the participant breakdown in Figure 2b, we observed that almost all participants (98.6% in the young group and 94.4% in the old group) have a better connectome prediction using congruent templates than using incongruent templates from the other group. Additional results predicting the fine-grained connectome for the middle-aged cohort using the middle-aged template are detailed in Appendix 1—figure 5. We also evaluated connectome prediction accuracies using templates constructed from 10-year age increments. The results show a continuous gradient of age-related divergence (Appendix 1—figure 6). When predicting data for the 80–90 cohort, the 20–30 template performs the worst, and the performance steadily improves as the template age gets closer to the target demographic. This systematic gradient further supports our main finding: the penalty for using an incongruent template increases with the discrepancy between the template age and participant age. The topographic distribution of mean differences between the predicted connectomes using congruent and incongruent templates is displayed in Figure 2c. Congruent templates generally perform better in the frontal and parietal lobes—regions primarily responsible for cognitive functions—which can be significantly influenced by age.

**Figure 3.**
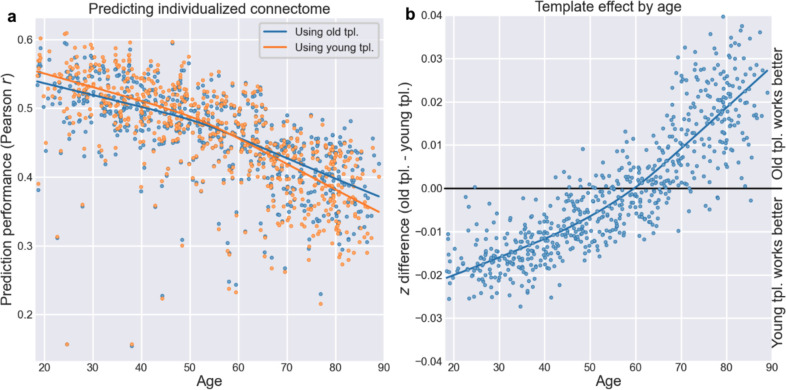
Comparison of individual connectome prediction across all age spans. (**a**) Scatter plot showing Pearson correlation between actual connectome and predicted connectome derived from young and old templates for individuals across all age spans. (**b**) Correlation difference among individuals between two age group templates, ordered by age. Figure 3—source data 1.Source data for comparison of individual connectome prediction across all age spans (Cam-CAN).

To evaluate the influence of age on the performance of hyperalignment template across the entire lifespan, we computed the correlation between actual connectome and predicted connectome of all participants using both young templates and old templates. As an individual’s age becomes more distant from the template age group, the relative performance of the template decreases (Figure 3a, b).

Overall, congruent age-specific templates better predict participants' connectomes than incongruent templates. As the participant’s age diverges further from the template age group, the prediction accuracy decreases. This result indicates that age-specific templates are necessary for improving the performance of hyperalignment.

### Predicting brain responses to the movie

**Figure 4.**
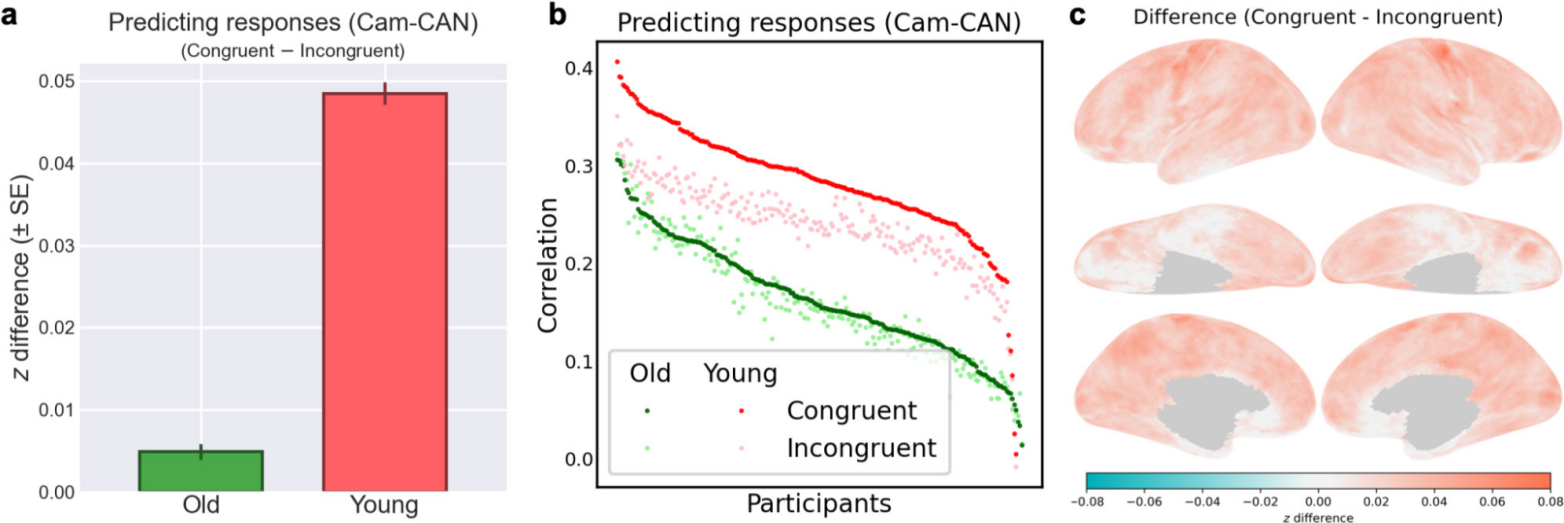
Predicting brain responses to the movie based on congruent and incongruent templates. (**a**) The average *z* (± SD) for the old group was 0.1661 ± 0.0600 for congruent templates, and 0.1612±0.0636 for incongruent templates. The mean difference (± SE) was 0.0049 ± 0.0010, *t*(212) = 5.02, Cohen’s *d* = 0.3443, and p < 10^–5^. The average *z* (± SD) for the young group was 0.2925 ± 0.0627 for congruent templates, and 0.2441 ± 0.0490 for incongruent templates. The mean difference (± SE) was 0.0485 ± 0.0013, *t*(209) = 35.99, Cohen’s *d* = 2.4833, and p < 10^–90^. (**b**) Scatter plot of each participant’s average predicted response values derived from different congruent and incongruent templates. (**c**) Topographic distribution of predicted response difference across different brain regions. Figure 4—source data 1.Source data for predicting brain responses to the movie based on congruent and incongruent templates (Cam-CAN).

Besides functional connectivity, congruent templates also performed better than incongruent templates in predicting neural responses to the movie (Figure 4). These results indicate that the more congruent the template age group is with a participant’s age, the more accurate the prediction becomes.

We used the same 10 functional templates for each age group, based on different participants randomly chosen as described earlier, to calculate individual transformation matrices and predict movie responses. We then focused on differences in performance between using congruent and incongruent templates.

Next, we computed correlation coefficients between the measured response time series to the movie and the predicted time series to evaluate template performance. The correlation coefficients were Fisher-transformed to *z* values and aggregated either across different parts of the brain (Figure 4a, b) or across participants (Figure 4c). Prediction performance for both groups was better when using congruent templates than incongruent templates, with the young group showing a larger congruency effect than the old group.

## Discussion

In this paper, we analyzed the effect of age on hyperalignment performance. Our findings demonstrate the significant impact of age-specific hyperalignment templates on the accuracy of individualized connectome prediction, neural response to the movie, as well as the ISC values after hyperalignment.

The ISC results demonstrate the age effect on hyperalignment template performance. Average ISC values in the hyperalignment common model space are higher when projecting individual connectome to the common model space derived from congruent age group templates—those aligned with the participant’s age group—rather than incongruent age group templates. By contrast, the congruency effect in the visual cortex is much smaller, suggesting that the aging effect on functional connectivity is smaller in these areas.

Moreover, congruent hyperalignment templates consistently achieved higher correlation between predicted individual connectomes and actual connectomes in participants’ native anatomical spaces compared to incongruent templates. When predicting an individual’s connectome in different age groups using either old group templates (>65 years old) or young group templates (<45 years old), as the individual’s age becomes more distant from the template age group, the prediction accuracy drops, and the difference between congruent and incongruent template prediction increases. Movie response prediction further indicates that the more congruent the template age group is with the participant’s age, the more accurate the prediction becomes.

We applied ISC analysis to an independent dataset, Dallas Lifespan Brain Study (DLBS), to further validate the generalizability of our findings (see Appendix 1). The results were consistent with those observed in the Cam-CAN dataset, showing that congruent templates yielded higher average ISC values than incongruent templates.

Previous hyperalignment study shows that the more data used in building the templates for estimating individual tuning matrices, the better the model performs (Feilong et al., 2023). We expect, therefore, that the effect of age-congruency of templates will be greater if the templates are built with larger datasets. Currently, the templates are built using only approximately 16 min of fMRI functional data from around 200 participants. We expect that better and more stable model performance could be achieved by incorporating data from more participants and increasing the amount of data per participant, preferably to at least an hour. Moreover, incorporating data using richer stimulus paradigms in template construction might provide additional information that could enhance template performance. Currently, our templates are constructed using only resting state and sensorimotor task data. Naturalistic stimuli, such as movie-viewing, more broadly sample cognitive and brain states. With increased data collection and richer, naturalistic stimulus paradigms, we could capture a broader range of neural activities and idiosyncrasies, potentially leading to more robust and flexible hyperalignment templates and a more precise and reliable alignment between individual connectomes and the common model space.

In this work, we only focused on the impact of age on hyperalignment templates and observed a significant improvement using age-congruent templates compared to incongruent ones. Based on these findings, it is important to consider the practical applications of this methodology in future research. When the goal of a case–control study is to directly compare functional organization or brain responses between distinct populations—such as clinical and non-clinical participants—it is essential that all individuals are hyperaligned to the same common template. For these analyses, researchers should either construct a joint template containing a balanced, representative sample from both groups, or align all participants to a normative control template based on age-matched data. This ensures that the resulting data share a single coordinate system, allowing for valid statistical comparisons between groups.

Age- or disease-specific templates are highly advantageous when the research objective is to maximize decoding accuracy or predictive performance within a specific population. Just as age introduces specific variations in neural architecture, neurological or psychiatric conditions—such as Alzheimer’s disease and Parkinson’s disease—can produce alterations in brain structure and function. In clinical or lifespan research, if the goal is to build a reliable biomarker of differences among individuals within a group, for example, of disease progression or disease subtypes, or to map individualized connectomes for a specific patient cohort, researchers should use a template congruent with that specific group. By constructing disease-specific templates, it may be possible to capture these distinctive neural patterns with greater accuracy, leading to more precise alignment of individual connectomes. This approach could provide insights into the pathophysiological mechanisms underlying various clinical conditions and broaden the use of hyperalignment models in clinical neuroscience.

## Methods

### fMRI dataset

The Cam-CAN dataset comprises fMRI data of over 600 people from a cross-sectional adult lifespan (18–87 years old) population-based sample, with approximately 25 min of fMRI data per individual (Taylor et al., 2017; Shafto et al., 2014). All MRI datasets were collected at MRC-CBSU using a 3T Siemens TIM Trio Scanner with a 32-channel head coil, 3.0 × 3.0 × 4.44 mm^3^ voxels and 20% gap. Each individual’s fMRI data were collected during three different tasks:

1. 8 min 40 s of resting state with eyes closed (‘rest’);
2. 8 min of movie-watching of the film ‘Bang! You’re dead’ (‘bang’);
3. 8 min 40 s of a sensorimotor task (‘smt’) during which participants were asked to press a button upon the presentation of a visual and/or auditory stimulus.

Both resting state and the sensorimotor task used single echo sequences with TR = 1970 ms, TE = 30 ms, flip angle = 78°, and the movie-watching task used multi-echo sequences with TR = 2470 ms, TEs = 9.4, 21.2, 33, 45, and 57 ms, flip angle = 78°. 646 participants have the full records of all three functional scans (age distribution shown in Table 1).

**Table 1.** Age group division and corresponding training/test set sizes.

Age group	Age range	Number of participants	Training group	Test group
Young	18–45	215	144	71
Old	65–90	216	144	72

We classified the participants into three different age groups—young, mid, and old—with each group consisting of approximately the same number of individuals. Our analyses focus on the young and old groups.

### Preprocessing

We preprocessed all MRI data using fMRIPrep (Esteban et al., 2019), with version 20.2.7. For each participant, the cortical surface was reconstructed using the high-resolution structural scans. The functional data were corrected for head motion, projected onto the cortical surface model, and resampled to the onavg-ico32 cortical surface template, which uniformly samples different parts of the cortex (Feilong et al., 2024). After that, we used linear regression to partial out nuisance regressors from our data. The list of nuisance regressors includes six head motion parameters and their derivatives, framewise displacement, global signal, six aCompCor components from cerebrospinal fluid and white matter, and polynomial trends up to the second order. After the regression, we normalized the time series of each cortical vertex to zero mean and unit variance.

### Individual connectome calculation

We computed two kinds of connectomes based on different functional scans:

1. using both resting state and sensorimotor task data,
2. using movie-watching data,

This division allows us to examine template performance using both functional connectivity and response time series to the movie. To calculate functional connectivity, we downsampled the data matrices for each individual and each task from ‘onavg-ico32’ space (19,341 vertices) to ‘onavg-ico8’ space (1210 vertices) and used the time series for downsampled data as connectivity targets. Correlations between connectivity target time series and vertex time series are indices of functional connectivity. Each vertex’s connectivity profile is a vector of 1210 elements, where each element is the correlation between the time series of the vertex and the time series of a connectivity target. We *z*-scored the connectivity profile for each of the 19,341 vertices, and these connectivity profiles collectively form the connectome matrix of the individual and task.

### Hyperalignment template creation

For each age group (young and old), we built the templates using approximately two-thirds of the participants and withheld the remaining one-third for testing. Given there are multiple ways to choose the participants, we repeated the procedure 10 times, each time randomly choosing two thirds of the participants without replacement. The participants used for evaluation were always independent from the participants used for training.

We combined fMRI data of different tasks to maximize the amount of data we use to compute the connectomes, given that differences in connectomes based on different tasks are much smaller in scale compared to individual differences (Gratton et al., 2018). Each whole-brain template was created using a searchlight-based algorithm (Feilong et al., 2023, section 4.2) with a 20-mm searchlight radius, based on the training group participants’ connectome data calculated from the resting state data and sensorimotor task data. There are 19,341 overlapping searchlights, each containing an average of approximately 121 vertices (range: 44–187). For each searchlight, we created a local template so that its representational geometry and topographies are representative of the training participants. We first concatenated the vertices of all participants and performed a PCA on the concatenated data, to derive a PC template that reflects the representational geometry of the searchlight, where *M*_(PC)_ is the PC template, *B*_(_*_p_*_)_ is the local data matrix of the *p*th participant, and *n* is the number of participants,

$$\displaystyle M_{(\mathrm{PC})} = \frac{1}{\sqrt{n}} \operatorname{PCA} \left( B_{(1)}, \ldots, B_{(n)} \right)$$

We then applied a rotational matrix *R* to the PC template to minimize the topographic differences without changing the information content, where $\parallel \cdot \parallel F$ is the Frobenius norm,

$$\displaystyle  R= \arg\,\mathop{\min}\limits_{R} \sum_{p=1}^{n} \left\| M_{(\mathrm{PC})}R - B_{(p)} \right\|_{F}^{2}$$

To find the solution *R*, we applied the orthogonal Procrustes algorithm to concatenated data matrices (see formula below).

$$\displaystyle R= \arg\mathop{\min}\limits_{R} \left\| \begin{bmatrix} B_{(1)}\\ \vdots\\ B_{(n)} \end{bmatrix} - \begin{bmatrix} M_{(\mathrm{PC})}\\ \vdots\\ M_{(\mathrm{PC})} \end{bmatrix} R \right\|_{F}^{2}$$

**Figure 5.**
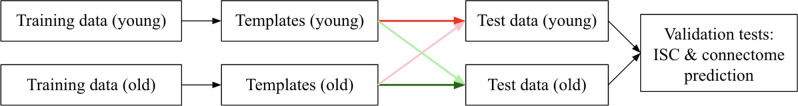
Schematic of the procedure for building and testing hyperalignment templates. Darker arrows indicate congruent templates (i.e., from the same age group), whereas light arrows indicate incongruent templates.

The local templates *M*_(PC)_*R* from all searchlights were then combined to form the whole-brain template. For each participant *p*, we derived two transformation matrices using the Procrustes algorithm: *R*_(_*_p_*_)_, which projects data from the participant’s native cortical space to the template space and was used in ISC analyses, and *R′*_(_*_p_*_)_, which projects data from the template space back to the participant’s native cortical space and was used in the prediction analyses (Figure 5, see also Appendix 1—figure 1). Note that these matrices differ from the rotation matrix used to derive the local template.

Given that functional connectomes may differ across tasks, for each template based on resting state and sensorimotor data, we also computed a corresponding template based on the movie-viewing data. This was used in the analysis of predicting individual connectomes, as described below.

### Inter-subject correlation

We computed the ISC of connectivity profiles in the common model space to evaluate template performance, where higher ISCs indicate better performance. For each participant in the test set, we computed the participant’s hyperalignment transformation matrix *R*_(_*_p_*_)_ using the connectome based on resting state and sensorimotor task data, and applied the transformation matrix to the original movie-watching responses to compute the hyperaligned connectome. This ensures that the data used to compute hyperalignment transformation are independent from the data used to evaluate performance. We computed ISC as the correlation between the participant’s connectivity profile and the average profile across other test participants in the same age group (Figure 5). Each participant has up to 20 ISC maps (10 young templates and 10 old templates), and we averaged ISC maps of the same kind (young or old) for each participant. We converted *r* values to *z* values (Fisher transform, arctanh(*r*)), averaged the *z* values, and converted them back to *r* values. Based on the analysis, we either averaged them across cortical vertices (Figure 1a, b, Appendix 1—figure 3a, b) or participants of the same age group (Figure 1c, Appendix 1—figure 3c).

### Predicting individual connectome

We also tested the prediction of connectomes in held-out test participants’ native anatomical space (Figure 5). We derived each participant’s predicted movie-viewing connectome as the matrix multiplication of the movie-viewing template and the individual’s transformation matrix *R′*_(_*_p_*_)_, derived from resting state and sensorimotor task data. We then compared the predicted connectome with the participant’s actual movie-viewing connectome based on the correlation of the corresponding connectivity profiles in these two connectomes.

### Predicting individual responses to the movie

Similarly, we predicted individual responses to the movie using the same templates created for the congruent and incongruent age groups. Using the Individualized Neural Tuning (INT) model (Feilong et al., 2023), each individual’s connectome was modeled as the same template connectome with an individualized transformation, and we established correspondence between the modeled connectome and the brain responses to the movie using the same group of training participants used to build the template. In other words, we applied the INT model to enrich the template so that it could predict individualized brain responses in addition to individualized connectomes based on the template. We then evaluated the template’s performance using correlation coefficients between the measured response time series to the movie and the predicted time series.

### Validation dataset

The results based on the Cam-CAN dataset demonstrate that hyperalignment templates from the same age group work better than templates from age-incongruent groups, and this effect generalizes across tasks (from resting state and sensorimotor to movie-watching) and scan protocols (from single- to multi-echo sequences). We further tested the generalizability of our results using an independent dataset—the DLBS dataset (Park et al., 2025), which was collected with a different scanner, a different group of participants, and different tasks (see Appendix 1).

## Figures and Tables

**Appendix 1—figure 1.**
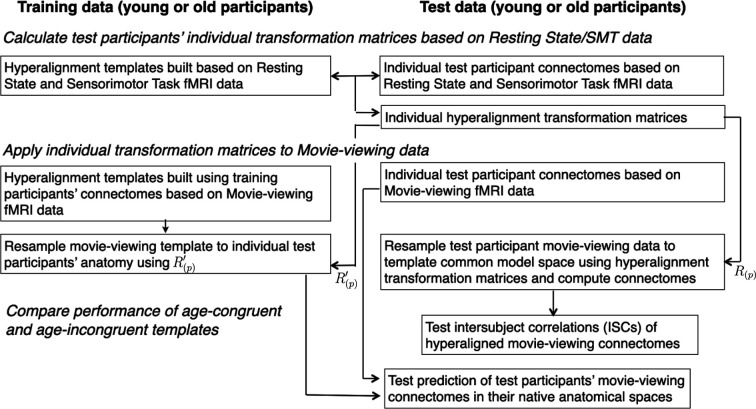
Schematic of the procedure for building and testing hyperalignment templates.

**Appendix 1—figure 2.**
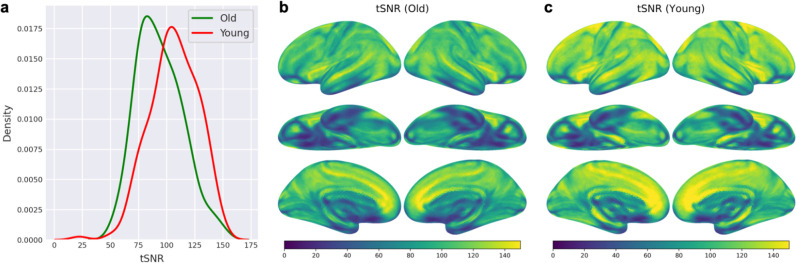
tSNR differences between age groups. (**a**) The distribution of tSNR across participants, separately for each age group. The distribution of tSNR across the cortex for the old group (**b**) and the young group (**c**). Appendix 1—figure 2—source data 1.Source data for tSNR differences between age groups.

**Appendix 1—figure 3.**
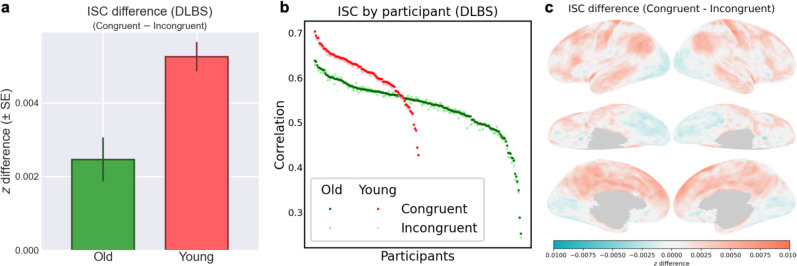
Inter-subject correlation results (DLBS). (**a**) The average *z* (± SD) for the old group was 0.6044 ± 0.0789 for congruent templates, and 0.6020 ± 0.0810 for incongruent templates. The mean ISC difference (± SE) was 0.0025 ± 0.0006, *t*(181) = 4.16, Cohen’s *d* = 0.3087, and p < 10^–4^. The average *z* (± SD) for the young group was 0.7087 ± 0.0827 for congruent templates, and 0.7034 ± 0.0809 for incongruent templates. The mean ISC difference (± SE) was 0.0053 ± 0.0004, *t*(91) = 13.24, Cohen’s *d* = 1.3806, and p < 10^–22^. (**b**) Scatter plot of each participant’s average ISC value derived from different congruent and incongruent templates. (**c**) Topographic distribution of ISC difference across different brain regions. Appendix 1—figure 3—source data 1.Source data for inter-subject correlation results (DLBS).

**Appendix 1—figure 4.**
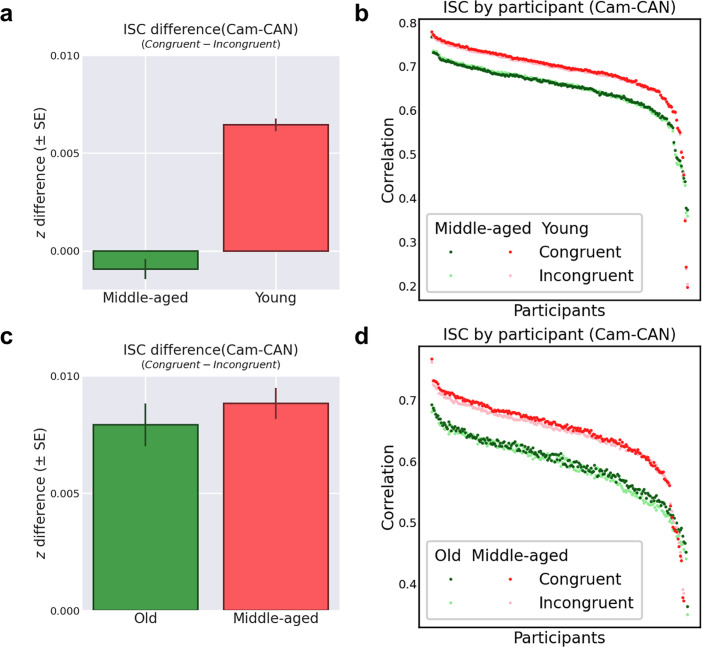
Inter-subject correlation results based on the middle-aged cohort (Cam-CAN). (**a**) Mean ISC difference (Congruent − Incongruent) for the middle-aged and young groups. The mean ISC difference (± SE) for the middle-aged group was −0.0009 ± 0.0005, while the mean ISC difference for the young group was 0.0064 ± 0.0003. (**b**) Scatter plot of each participant’s average ISC value derived from congruent and incongruent templates for the middle-aged versus young comparison. (**c**) Mean ISC difference (Congruent − Incongruent) for the old- and middle-aged groups. The mean ISC difference (± SE) for the old group was 0.0079 ± 0.0009, and the mean ISC difference for the middle-aged group was 0.0088 ± 0.0007. (**d**) Scatter plot of each participant’s average ISC value derived from congruent and incongruent templates for the old- versus middle-aged comparison. Appendix 1—figure 4—source data 1.Source data for inter-subject correlation results based on the middle-aged cohort (Cam-CAN).

**Appendix 1—figure 5.**
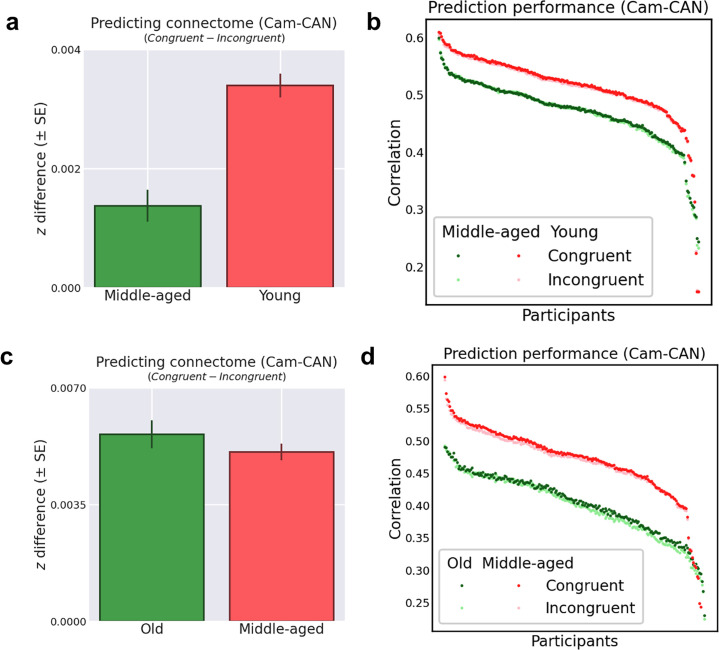
Connectome prediction results based on the middle-aged cohort (Cam-CAN). (**a**) Mean *z*-difference (Congruent − Incongruent) in connectome prediction accuracy for the middle-aged and young groups. The mean difference (± SE) for the middle-aged group was 0.0014 ± 0.0003, while the mean difference for the young group was 0.0034 ± 0.0002. (**b**) Scatter plot of each participant’s connectome prediction correlation derived from congruent and incongruent templates for the middle-aged versus young comparison. (**c**) Mean *z*-difference (Congruent − Incongruent) in connectome prediction accuracy for the old- and middle-aged groups. The mean difference (± SE) for the old group was 0.0056 ± 0.0004, and the mean difference for the middle-aged group was 0.0051 ± 0.0002. (**d**) Scatter plot of each participant’s connectome prediction correlation derived from congruent and incongruent templates for the old- versus middle-aged comparison. Appendix 1—figure 5—source data 1.Source data for connectome prediction results based on the middle-aged cohort (Cam-CAN).

**Appendix 1—figure 6.**
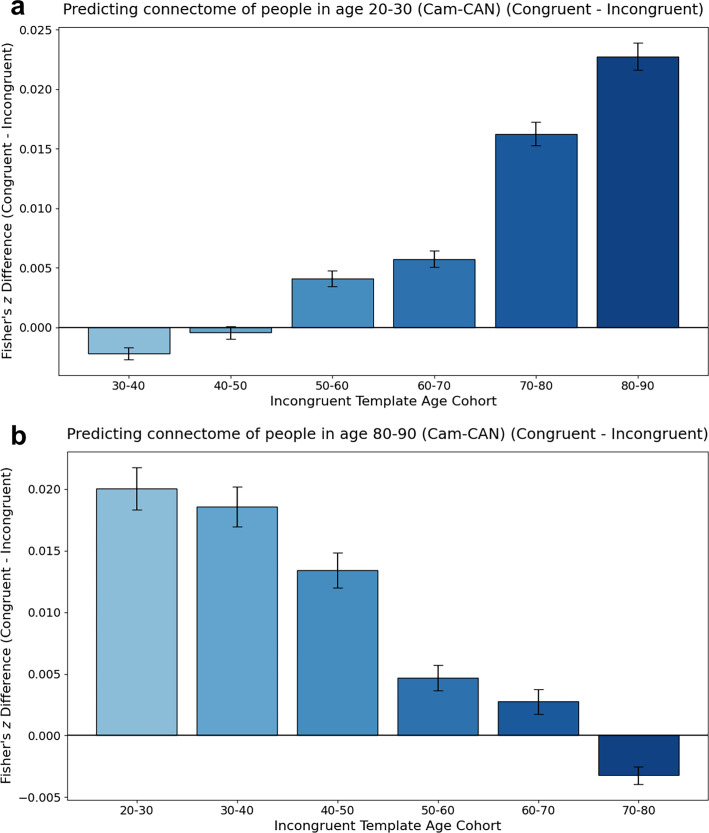
Connectome prediction results with 10-year age templates: (**a**) Mean Fisher’s *z*-difference (Congruent − Incongruent) in connectome prediction for the young cohort (20–30 years old). The bar plot compares the performance of the age-congruent template against incongruent templates from progressively older cohorts (ranging from 30–40 to 80–90). (**b**) Mean Fisher’s *z*-difference (Congruent − Incongruent) in connectome prediction for the older cohort (80–90 years old), comparing the age-congruent template against incongruent templates from progressively younger cohorts (ranging from 20–30 to 70–80). Appendix 1—figure 6—source data 1.Source data for connectome prediction results with 10-year age templates.

## Data Availability

We have used two previously published datasets: Cambridge Centre for Ageing and Neuroscience (Cam-CAN) dataset (https://opendata.mrc-cbu.cam.ac.uk/projects/camcan/) and the Dallas Lifespan Brain Study (DLBS) dataset (https://openneuro.org/datasets/ds004856). All code used for data preprocessing, analysis, and figure generation is available on GitHub at: https://github.com/yuqi98/Aging_templates_scripts/ (copy archived at Zhang and Ma, 2026). The following previously published dataset was used: ParkD HennesseeJ SmithET ChanM KatenC BacciJ FrankS MonierS CollyerA TammingaC MooreW RofskyN RodrigueK KennedyK WigG 2025The Dallas Lifespan Brain StudyOpenNeuro10.18112/openneuro.ds004856.v1.3.0

## Additional files

MDAR checklist

Supplementary file 1. All subgraphs regenerated using the minimal data files.

Source data 1. Code to regenerate all graphs from the data files provided.

## Appendix 1

### Group differences in data quality

In the main analysis, we found that both inter-subject correlation (ISC) and prediction accuracy were higher for the young group than the old group, regardless of template type. We suspect that this might be related to data quality differences between the groups. Therefore, we performed an additional analysis, computing the temporal signal-to-noise ratio (tSNR) as a data quality measure, and we compared it between the two groups. We computed tSNR as the mean signal divided by the standard deviation for each participant and each vertex, using the resting-state data from the Cam-CAN dataset. We found that in general the average tSNR for the young group (mean ± standard deviation = 106.4 ± 21.2) is higher than the old group (93.9 ± 20.3), *t*(429) = 6.25, p < 10^–9^.

### Validation dataset—Dallas Lifespan Brain Study

The Dallas Lifespan Brain Study (DLBS) dataset collects multi-modal data from over 400 individuals across the entire adult lifespan, ranging from 20 to 90 years old. The dataset contains longitudinal data from multiple scan sessions conducted over 10 years. In this study, we focused on the fMRI data from the first and most complete scan session, which was collected between 2008 and 2014 from 464 participants. The fMRI data were collected using a Philips Achieva 3T scanner at the University of Texas Southwestern Medical Center, with 3.4 × 3.4 × 3.5 mm^3^ voxels, TR = 2000 ms, TE = 25 ms, flip angle = 80° (Chan et al., 2014; Chen et al., 2021; Kennedy et al., 2015; Park et al., 2012). Among these participants, 407 completed a scanning session that included: (1) 7 min and 56 s of the words task, during which participants were shown 128 words and asked to make a semantic judgment on whether each word represented something living or nonliving; (2) three 5 min and 56 s runs of the scenes task, where participants were presented with outdoor landscape scenes and had to determine whether water was present in each scene; and (3) 6 min and 14 s of the resting state. Among the 407 selected participants, 92 were younger than 45 years old, 133 were between 45 and 65 years old, and 182 were older than 65 years old.

The DLBS dataset was preprocessed using fMRIPrep version 24.1.0. Consistent with the procedure applied to the Cam-CAN dataset, we computed two types of functional connectomes based on different scan conditions:

1. using DLBS resting state and words task data,
2. using DLBS scenes task data.

### Inter-subject correlation

We applied the hyperalignment templates—constructed using Cam-CAN data from the two age groups (young and old)—to the DLBS dataset. Since the two datasets do not share identical functional tasks, we calculated transformation matrices that resample the resting state and words task fMRI data of the DLBS dataset into the Cam-CAN templates for young and old participants. We then applied the transformation matrices to connectomes based on the scenes task data and calculated the ISCs. The ISC results align well with those from the Cam-CAN dataset (Figure 3). We observed a statistically significant difference between ISC values derived from congruent and incongruent templates in both age groups (Appendix 1—figure 3a). Although the differences among individual participants were small, they were still statistically significant with an anatomical distribution that is consistent with the congruency effects in the Cam-CAN dataset (Appendix 1—figures 3b, 92.4% participants in the young group and 57.7% participants in the old group have higher congruent ISC values). Similar to the results with the Cam-CAN data, we noted a stronger effect of template congruency on ISCs in the parietal, temporal, and frontal cortices, with smaller effects in occipital and ventral temporal visual cortices (Appendix 1—figure 3c).

### ISC Cam-CAN middle-aged template

We further evaluated the effect of age congruency by introducing an intermediate middle-aged cohort to our Cam-CAN dataset analysis. We computed the *z*-difference between ISC values after hyperalignment derived from congruent and incongruent templates to compare the middle-aged group against both the young and old cohorts (Appendix 1—figure 4). When comparing the middle-aged group to the young cohort, aligning middle-aged participants to their strictly age-congruent template actually resulted in slightly lower overall ISCs compared to using the young incongruent template (Appendix 1—figure 4a). However, the young group maintained a strong congruency advantage over the middle-aged template. Conversely, when comparing the old cohort with the middle-aged cohort, both groups exhibited higher ISCs when aligned to their respective congruent templates (Appendix 1—figure 4c). Breaking down the data by individual confirmed these distinct group-level trends across the majority of participants (Appendix 1—figure 4b, d).

### Predicting connectomes Cam-CAN middle-aged template

We also evaluated the impact of age congruency on fine-grained connectome prediction accuracy by incorporating the intermediate middle-aged cohort. We computed the *z*-difference between predicted and actual connectome correlations derived from congruent and incongruent templates. In contrast to the ISC results, connectome prediction demonstrated a consistent congruency advantage across all age comparisons. When comparing the middle-aged cohort to the young cohort, both groups exhibited significantly higher prediction accuracies when aligned to their respective age-congruent templates (Appendix 1—figure 5a). Similarly, when comparing the old cohort with the middle-aged cohort, predictions generated using congruent templates were consistently more accurate than those using incongruent templates for both groups (Appendix 1—figure 5c). Examining the breakdown by individual participants confirmed these robust group-level trends, illustrating that the vast majority of participants across all age groups achieved optimal connectome prediction when aligned to a template congruent with their specific age cohort (Appendix 1—figure 5b, d).

### Predicting connectomes Cam-CAN with 10-year age templates

We have constructed templates using narrower, 10-year age intervals and evaluated their performance. Because different age groups have different numbers of people, we use a fixed number of training participants for each age group (two-thirds of the people from the group with the minimal number of people) to build the templates to make a fair comparison. The results show a continuous gradient of age-related divergence. When predicting data for the 80–90 cohort, the 20–30 template performs the worst, and the performance steadily improves as the template age gets closer to the target demographic. This systematic gradient further supports our main finding: the penalty for using an incongruent template increases with the discrepancy between the template age and participant age.
