# Supplementary material for: Boosting hyperalignment performance with age-specific templates
Source: eLife. 2026 Aug 5;15:RP110566. doi: 10.7554/eLife.110566 (PMC13441295; doi:10.7554/eLife.110566)
Supplement: Peer review history

# Editor report: eLife Assessment

**DOI:** 10.7554/eLife.110566.3.sa0

**Authors:** Shuo Wang

**Affiliations:** Washington University in St. Louis United States

**Keywords:** Convincing, Valuable

This **valuable** study advances our understanding of best practices for analyzing population-level data using advanced functional alignment methods. It provides **convincing** evidence that demographic-specific functional templates improve functional neuroimaging studies that use hyperalignment. This study will be of interest to cognitive neuroscientists, neuroimaging methodologists, and computational researchers with an interest in the human brain.

# Referee report: Reviewer #1 (Public review)

**DOI:** 10.7554/eLife.110566.3.sa1

The authors present a compelling case for the necessity of age-specific templates in functional hyperalignment. Given that the brain undergoes substantial developmental, structural, and functional changes across the lifespan, a 'one-size-fits-all' canonical template is often insufficient. This study effectively demonstrates that incorporating age-congruent features significantly enhances the performance and sensitivity of hyperalignment models. By validating these findings across two independent datasets (Cam-CAN and DLBS), the paper provides robust evidence that accounting for age-related functional organization is a critical prerequisite for accurate functional alignment in lifespan research

Comments on revised version:

The authors have been exceptionally thorough in addressing the concerns raised by the reviewers. In particular, the inclusion of the supplemental analysis on the middle-aged cohort is a valuable addition that strengthens the manuscript. Furthermore, the rationale for employing a congruent template is well-articulated; this approach clearly provides a more robust and accurate foundation for reconstructing individualized connectomes. I appreciate the authors' detailed responses and have no further comments.

# Referee report: Reviewer #2 (Public review)

**DOI:** 10.7554/eLife.110566.3.sa2

Summary:

In this study, Zhang and colleagues examine the role of participant selection in creating and using functional templates to improve analyses using hyperalignment. Hyperalignment aligns participants' functional MRI data to a shared functional template, analogous to the anatomical templates used to bring anatomical MRI data into a shared space (e.g., MNI152). The question of appropriate template creation is especially pressing for population-level analyses, where a large number of demographic groups (e.g., different age ranges, clinical statuses) may be included in the same analysis. These different demographic groups may have differences in their functional organization that complicate the creation of a single study-specific functional template.

To provide an initial investigation of the potential effect of demographic-specific templates, the authors use the publicly available Cam-CAN dataset which contains participants from 18 to 87 years of age. They define a young adult (< 45 years of age) and an older adult group (> 65 years of age) from this dataset with approximately the same number of participants. They investigate whether "age-congruent" templates (i.e. defined in the same age group they are used) improve three analyses where hyperalignment has been previously shown to boost performance: inter-subject correlation, predicting individual connectomes, and predicting individual functional responses. Using the Cam-CAN derived older adult template, they then replicate the ISC analyses using the publicly available Dallas Lifespan Brain Study (DLBS).

Overall, the presented results are highly suggestive that age-congruent templates consistently improve performance, though the absolute effects are small.

Strengths:

The use of a separate validation sample-re-using the same template calculated with Cam-CAN-highlights the potential of developing independent templates for individual demographic groups and then distributing these for wider use, analogous to the MNI templates that are widely used throughout the field of neuroimaging. This suggests that the potential impact of this framework is significant.

Weaknesses:

In their revision, the authors have addressed the previously raised "weaknesses" by providing guidance for researchers interested in using age-specific hyperalignment templates in practice.

Impact:

Overall, this work is likely to encourage future development of age-specific functional templates in the imaging community.

# Author comment: Author response

**DOI:** 10.7554/eLife.110566.3.sa3

**Authors:** Yuqi Zhang, M Ida Gobbini, James V Haxby, Ma Feilong

**Affiliations:** Dartmouth College Hanover United States; University of Bologna Bologna Italy; Dartmouth College Hanover United States; University of South Carolina Columbia United States

The following is the authors’ response to the original reviews.

**Public Reviews:**

**Reviewer #1 (Public review):**

Summary:

The authors present a compelling case for the necessity of age-specific templates in functional hyperalignment. Given that the brain undergoes substantial developmental, structural, and functional changes across the lifespan, a 'one-size-fits-all' canonical template is often insufficient. This study effectively demonstrates that incorporating age-congruent features significantly enhances the performance and sensitivity of hyperalignment models. By validating these findings across two independent datasets (Cam-CAN and DLBS), the paper provides robust evidence that accounting for age-related functional organization is a critical prerequisite for accurate functional alignment in lifespan research.

Strengths:

(1) The authors used three metrics to evaluate performance. Across all metrics, they found that age-congruent templates outperformed age-incongruent templates, suggesting that age-specific templates can improve alignment.

(2) These findings highlight the superiority of age-congruent templates for hyperalignment. This work underscores the importance of age-matching in cross-subject functional mapping and represents a vital step forward for the methodology.

We thank the reviewer for the summary and the positive evaluation of our manuscript.

Weaknesses:

(1) Participant Demographics and Group Separation:

The study defines the 'older' cohort as 65-90 years and the 'younger' cohort as 18-45 years. While this 20-year gap (ages 46-64) effectively maximizes the contrast between groups, the results in Figure 4a suggest that the predicted individualized connectomes follow a continuous distribution. Given this continuity, could the authors provide the average median trends for Figures 2a and 2b to illustrate how the model behaves across the missing age range?

Thanks for raising this important point. We had calculated the results for the middle-aged cohort template and have included them in the Supplementary Figures 4 & 5. Similar to Figure 2a, 2b, 3a and 3b, we directly compare the intersubject correlation and prediction performance of the middle-aged participants when aligned to their congruent middle-aged template versus an incongruent template. We observed consistent results across validation analyses (ISC and prediction) and groups (young vs. middle-aged, middle-age vs. old). Consistent with our main findings, the middle-aged cohort exhibits significantly higher intersubject correlation and prediction performance when using the age-congruent middle-age template. These results confirm that the age-related shifts in functional brain organization captured by the hyperalignment templates follow a continuous trajectory across the lifespan.

(2) Request for Implementation:

I have been unable to locate the source code associated with this publication. Could the authors please provide a link to the repository or clarify if the implementation is available for reproduction?

We have made our scripts public in GitHub and here’s the link: https://github.com/yuqi98/Aging_templates_scripts

(3) Analysis of Prediction Performance and Distribution:

While Figures 3b and 5b clearly demonstrate that the congruent template improves correlation, Figure 4a shows a distinct shift in the scatter distribution. Could the authors provide a detailed explanation of the prediction performance metrics used? Specifically, I would like to understand how the underlying method accounts for the distribution differences observed when applying the congruent template.

Our prediction performance metric is the average Pearson correlation. We calculated the correlation between the model-predicted data (the individualized connectome in Figure 3 and the movie response in Figure 5) and the participant's actual measured data for each cortical vertex and averaged the correlations across vertices. A higher correlation indicates that the group template, when combined with the participant’s individualized transformation matrix, more accurately reconstructs the individualized functional connectome and responses to stimuli.

The distinct upward shift in prediction performance when using a congruent template occurs because brain functional organization shows age-specific features. A congruent template captures these age-specific connectivity and response features. Importantly, the template creation algorithm aims to reflect the central tendency of the training data, including representational/connectivity geometry and functional topographies. Therefore, the observed differences in templates reflect differences in functional organization across age groups. As a result, when projecting the common template back into an individual’s native cortical space using the transformation matrix derived from independent data, the congruent template provides a richer, more accurate basis for reconstructing the individualized connectome and movie-watching responses.

**Reviewer #2 (Public review):**

Summary:

In this study, Zhang and colleagues examine the role of participant selection in creating and using functional templates to improve analyses using hyperalignment. Hyperalignment aligns participants' functional MRI data to a shared functional template, analogous to the anatomical templates used to bring anatomical MRI data into a shared space (e.g., MNI152). The question of appropriate template creation is especially pressing for population-level analyses, where a large number of demographic groups (e.g., different age ranges, clinical statuses) may be included in the same analysis. These different demographic groups may have differences in their functional organization that complicate the creation of a single study-specific functional template.

To provide an initial investigation of the potential effect of demographic-specific templates, the authors use the publicly available Cam-CAN dataset, which contains participants from 18 to 87 years of age. They define a young adult (< 45 years of age) and an older adult group (> 65 years of age) from this dataset with approximately the same number of participants. They investigate whether "age-congruent" templates (i.e. defined in the same age group they are used) improve three analyses where hyperalignment has been previously shown to boost performance: inter-subject correlation, predicting individual connectomes, and predicting individual functional responses. Using the Cam-CAN-derived older adult template, they then replicate the ISC analyses using the publicly available Dallas Lifespan Brain Study (DLBS).

Overall, the presented results are highly suggestive that age-congruent templates consistently improve performance, though the absolute effects are small.

Strengths:

The use of a separate validation sample, reusing the same template calculated with Cam-CAN, highlights the potential of developing independent templates for individual demographic groups and then distributing these for wider use, analogous to the MNI templates that are widely used throughout the field of neuroimaging. This suggests that the potential impact of this framework is significant.

We thank the reviewer for the summary and the positive evaluation of our manuscript.

Weaknesses:

While the authors appropriately highlight the potential applications of this result (e.g., to different clinical statuses), it is not apparent how to appropriately extend this methodology to many common experimental paradigms. For example, in case-control studies (where researchers are interested in comparing clinical and non-clinical participants) the use of two different functional templates may complicate rather than ease analyses. Providing this as a potential limitation of the current template construction method, or providing recommendations to researchers interested in comparing across groups, would help to increase the impact of this work.

We appreciate the reviewer raising this important practical consideration. We have added additional explanation to the Discussion section to provide clear recommendations for researchers applying this methodology, which we summarize below:

When the goal of a case-control study is to directly compare functional organization or brain responses between clinical and non-clinical participants, it is essential that all individuals are hyperaligned to the same common template. For these analyses, researchers should either construct a joint template containing a balanced, representative sample from both groups, or align all participants to a normative control template. This ensures that the resulting data share a single coordinate system, allowing for valid statistical comparisons between groups.

However, disease-specific or age-specific templates are highly advantageous when the research objective is to maximize decoding accuracy or predictive performance within a specific population. In real world clinical or lifespan research, if the goal is to build a reliable diagnostic biomarker for disease progression or map individualized connectomes for a specific patient's cohort, researchers should use a template congruent with that specific group. The congruent template will preserve the group-specific representational geometry, providing a better individual-level prediction than a general cortical template.

**Recommendations for the authors:**

**Reviewer #2 (Recommendations for the authors):**

In general, there appears to be significantly more spread in the values for older adults (e.g., Figure 4b). It would be useful to know whether subdividing this group improves its relative performance; however, this will likely require additional investigation into the number of participants needed to establish a minimal template.

We thank the reviewer for this constructive comment. We agree that older adults exhibit greater inter-individual variability in functional organization, which likely drives the larger spread observed in Figure 4b. We also appreciate the suggestion to subdivide this group to see if narrower age bins improve relative performance.

We have constructed templates using narrower, 10-year age intervals and evaluated their performance. Because model performance increases with the amount of training data, we use a fixed number of training participants for each age group (two thirds of the people from the group with the minimal number of people) to build the templates to make a fair comparison. We have added the results in the Supplementary Figure 6. The results show a continuous gradient of age-related divergence. When predicting data for the 80–90 cohort, the 20–30 template performs the worst and the performance steadily improves as the template age gets closer to the target demographic. This systematic gradient further supports our main finding: the penalty for using an incongruent template increases with the discrepancy between the template age and participant age.

Interestingly, we noticed that at the extreme ends of the age range (20–30 and 80–90), the strictly congruent template was slightly outperformed by the immediately adjacent age bin (i.e., the 30–40 template for young participants, and the 70–80 template for the oldest participants). Because we strictly matched the number of training subjects across all bins, this slight dip is likely driven by differences in raw data quality. It is common for fMRI data from the extreme ends of the lifespan to have slightly lower signal-to-noise ratios or higher head motion compared to the intermediate 30–40 or 70–80 cohorts. This suggests that while age congruency is a key driver of hyperalignment success, the intrinsic data quality of the cohort used to build the template also plays a practical role in its overall performance.

This brings up the reviewer’s second point regarding the number of participants needed to establish a minimal template. Subdividing the age groups reduces the sample size available to construct each template. Previous research has demonstrated that while a hyperalignment template derived from a relatively small number of participants can achieve acceptable performance, increasing the amount of data and the number of subjects in the template space consistently and robustly improves alignment quality (See Supplementary Figure 7 in Feilong et al., 2023). Ultimately, our long-term goal is to build highly robust, standardized templates for fine-grained age cohorts across the entire lifespan. We are preparing to collect large-scale datasets from age 20 to 100 to build age-specific templates and provide them as open resources. This will allow future researchers to directly align their data to an age-appropriate template without needing to construct one from their own limited samples.

Reference

Feilong, M., Nastase, S. A., Jiahui, G., Halchenko, Y. O., Gobbini, M. I., & Haxby, J. V. (2023). The individualized neural tuning model: Precise and generalizable cartography of functional architecture in individual brains. Imaging Neuroscience, 1, 1–34. https://doi.org/10.1162/imag_a_00032
